# Faba Bean (*Vicia faba* L.) Albumin Isolate: Properties, Production and Potential Applications in Food Supplements and Medical Nutrition

**DOI:** 10.3390/foods15213840

**Published:** 2026-10-27

**Authors:** Patricija Radoš, Ekaterina Šprajc, Antun Jozinović, Slaven Vajnaht, Đurđica Ačkar

**Affiliations:** 1 Faculty of Food Technology Osijek, Josip Juraj Strossmayer University of Osijek, Franje Kuhača 18, 31000 Osijek, Croatia; ajozinovic@ptfos.hr (A.J.); dackar@ptfos.hr (Đ.A.); 2 MAR&E Experience Design d.o.o., Raška ulica 29, 10000 Zagreb, Croatia; esprajc@mare-experience.com; 3 NutriS d.o.o., Varaždinska 68, 33520 Novi Senkovac, Croatia; svajnaht@nutris.hr

**Keywords:** faba bean, albumin fraction, albumin isolate, food supplements, medical nutrition

## Abstract

Faba bean (*Vicia faba* L.) is a valuable source of plant proteins, with albumins representing a less abundant, water-soluble protein fraction. The aim of this review is to present the composition and properties of the albumin fraction of faba bean and to assess the potential for developing an albumin isolate for use in food supplements and medical nutrition. The review covers isolation and purification methods, amino acid composition, digestibility, functional properties and safety aspects. The available results suggest that the albumin fraction may have a favourable amino acid composition, good solubility and properties relevant to its use in powdered and liquid products. However, reported values vary depending on the raw material and the isolation and analytical methods used. Particular attention should be given to the potential presence of protease inhibitors, lectins, vicine and convicine, as well as to the insufficiently studied allergenicity of isolated albumins. Previous studies have mainly been conducted under laboratory conditions and studies directly investigating the faba bean albumin fraction remain limited. Based on the available knowledge, faba bean albumin isolate shows potential as a plant protein ingredient, but its properties and possible advantages over existing protein sources still need to be confirmed using a well-characterised isolate and in specific product formulations.

## 1. Introduction

The growth of the global population, changes in dietary habits and the need to reduce the environmental impact of food production have increased interest in developing more sustainable food sources. As the production of animal-based foods requires considerable amounts of natural resources and is associated with greenhouse gas emissions, plant protein sources are increasingly considered as an option for developing a more sustainable food system [1]. Growing consumer interest in a balanced diet, physical activity and maintaining good health has also contributed to the increasing presence of protein-enriched products in the diet [2]. At the same time, there is a need to develop food supplements and medical nutrition products for people with specific nutritional needs [3]. When selecting new protein sources, not only the protein content of the raw material is important, but also the possibility of producing protein preparations with suitable nutritional quality and properties for their intended use [4].

Among plant protein sources, legumes are particularly important as they provide proteins, complex carbohydrates, dietary fibre, vitamins, minerals and various bioactive compounds, including phenolic compounds, carotenoids, alkaloids, saponins and phytosterols. Soybean and pea are among the most widely used sources of plant protein preparations, so the use of less exploited legumes could broaden the range of protein sources and contribute to the diversity and sustainability of the food system. In this context, faba bean (*Vicia faba* L.) is an interesting raw material due to its high protein content, adaptability to different growing conditions and ability to fix atmospheric nitrogen through symbiosis [1,4,5].

Faba bean is one of the oldest cultivated legumes and its dry seeds generally contain approximately 20–35% protein, 40% starch and 1–2% fat. Total dietary fibre in whole seeds has been reported at approximately 15–27% on a dry matter basis. Faba bean proteins have a favourable amino acid composition, particularly due to their high lysine content, while methionine and cysteine are present in lower amounts [4,6,7,8]. However, the nutritional value of faba bean proteins depends not only on their amino acid composition, but also on their digestibility and the changes that may occur during isolation and processing. Therefore, when developing a protein isolate, its composition, production method and properties relevant to its use in food products should all be considered [8,9].

Globulins are the predominant protein fraction in faba bean, while albumins are present in smaller amounts. Albumins differ from globulins in their water solubility, protein profile and amino acid composition. They may contain higher levels of sulphur-containing amino acids, which are often limiting in legume proteins, while their good solubility may be useful in beverages, powdered preparations and other products where good protein dispersion and stability are important [8,10]. The separately isolated albumin fraction of faba bean has been studied considerably less than globulins and total protein isolates. Available data indicate its potential, but are still insufficient for a reliable assessment of the properties and possible applications of faba bean albumin isolate in food supplements and medical nutrition [1,10,11].

Previous reviews have addressed different aspects of faba bean proteins. Martineau-Côté et al. [1] reviewed their nutritional quality and potential health-related activities, with particular emphasis on protein-derived bioactive peptides and the effects of processing. They reported that appropriate processing can improve protein digestibility, while highlighting the need for further evidence to establish the relevance of the reported bioactivities in humans. Sharan et al. [12] focused on the production of faba bean flours, concentrates and isolates, explaining how processing changes ingredient functionality through alterations in composition and protein structure. Their review also identified flavour, colour and antinutritional factors as important constraints on food applications. Augustin and Cole [4] considered faba bean protein within the broader context of sustainable food systems and argued that ingredient development requires coordinated decisions across crop production, processing, nutrition and consumer acceptance. These reviews address faba bean protein ingredients broadly, with less attention to the separately recovered albumin fraction. The present review focuses on this fraction, distinguishing findings obtained directly for albumins from those reported for whole seeds, mixed protein preparations and hydrolysates.

The aim of this review is to summarise and critically evaluate current knowledge on the albumin fraction of faba bean in relation to the development of an isolate for food supplements and medical nutrition. It examines isolation methods, composition, nutritional quality, physicochemical and functional properties and safety considerations. Particular attention is given to whether the available evidence supports potential advantages over conventional globulin-rich faba bean isolates and to the research needed to assess suitability for the intended applications.

## 2. Literature Search Strategy

The literature included in this review was identified through searches of the Scopus, Web of Science and PubMed databases conducted between August and September 2026. The search was based on terms related to faba bean proteins and the albumin fraction, including “faba bean protein”, “Vicia faba protein”, “faba bean albumin”, “faba bean albumin fraction”, “faba bean albumin isolate” and “faba bean protein fractions”. The terms were used individually and in different combinations using the Boolean operators AND and OR.

The main search terms were additionally combined with expressions related to protein extraction and purification, including “protein extraction”, “protein isolation”, “protein purification”, “aqueous extraction”, “Osborne fractionation”, “isoelectric precipitation”, “membrane filtration”, and “ultrafiltration”. Literature addressing composition, nutritional value and functional properties was identified using terms such as “amino acid composition”, “protein profile”, “digestibility”, “protein quality”, “solubility”, “foaming properties”, “emulsifying properties” and “gelation”. Safety-related searches combined the main terms with “antinutritional compounds”, “protease inhibitors”, “lectins”, “vicine”, “convicine”, “allergenicity” and “food safety”. Potential applications were explored using the terms “food supplements”, “medical nutrition”, “foods for special medical purposes” and “oral nutritional supplements”. Relevant official European Union regulations and regulatory documents were additionally consulted to assess the requirements related to the potential applications of the isolate.

The identified publications were initially screened by title and abstract. Articles considered relevant to the aim and scope of the review were subsequently assessed in full text. The reference lists of relevant original research articles and review papers were also examined to identify additional publications, which were assessed using the same relevance criteria. Original research articles and review papers published in English were included when they addressed faba bean proteins, protein fractions, the isolation and properties of the albumin fraction, nutritional value, digestibility, functional properties, safety, or potential food applications. Priority was given to recent publications and studies directly investigating the faba bean albumin fraction. As the number of such studies remains limited, relevant research on total faba bean protein isolates and concentrates, as well as comparative studies of albumins from other pulses, was also considered. During interpretation, findings obtained directly for the albumin fraction were distinguished from those reported for total protein preparations. Publications dealing exclusively with crop production, plant diseases, cellular physiology or livestock performance were outside the scope of the review. Findings from broader studies were considered when they provided information relevant to the composition, nutritional quality or safety of food protein ingredients. No formal risk-of-bias or evidence-quality assessment was performed because the paper was designed as a narrative literature review.

The narrative synthesis draws on 60 references, comprising 51 scientific articles, 8 European Union legislative documents and one FAO report. The 51 scientific articles cited in the review were classified by publication year (Table 1) and principal subject (Table 2) to describe the literature used in the synthesis. Legislative documents and the FAO report were excluded from the publication-year and thematic distributions. Each article was assigned to one principal thematic category according to its main contribution to the review, although individual articles could address more than one topic.

25 of the 51 scientific articles (49%) were published between 2024 and 2026, including 9 in 2024, 11 in 2025 and 5 in 2026. Extraction and fractionation formed the largest thematic category, with 10 articles. Background and previous reviews and physicochemical and functional properties each accounted for 8 articles, while food applications and clinical nutrition context accounted for 9. Composition and nutritional value and digestibility and bioactivity each accounted for 5 articles, while 6 articles addressed antinutritional compounds and safety. These distributions describe the literature cited in this review rather than publication trends across the entire field.

The cited literature provides a broad context for evaluating faba bean proteins, but evidence directly addressing the separately isolated albumin fraction remains limited. The contribution of this review lies in bringing together findings on albumin composition, isolation, functional properties, nutritional quality and safety to assess its potential use in food supplements and medical nutrition. Evidence from total protein preparations and other pulses provides supporting context, while being distinguished from findings obtained directly for faba bean albumins.

## 3. Faba Bean as a Source of Plant Protein

Faba bean (*Vicia faba* L.) belongs to the Fabaceae family. It has been part of the diet in Europe, Asia and North Africa for centuries, particularly in the Mediterranean region, where it is consumed fresh, dried and processed [1,13]. Its adaptability to different climatic conditions makes it an interesting raw material for the local production of plant protein. Faba bean is able to fix atmospheric nitrogen through symbiosis, which reduces the need for mineral nitrogen fertilisers and contributes to more sustainable agricultural production. Despite its long history of cultivation and favourable nutritional value, its use in the food industry is still less common than that of soybean and pea [1,4].

In recent years, interest in faba bean has increased due to the need to include new plant protein sources in the food system. Its use is no longer limited to whole seeds and traditional dishes, but has expanded to the production of flour, protein concentrates and isolates, as well as protein-enriched products [6,7]. The possibility of separating individual protein fractions further broadens its potential applications, as it allows the production of preparations suited to different nutritional and technological requirements. For this reason, faba bean is considered a potential raw material for the production of functional food products, high-protein formulations and alternatives to animal-based products.

### 3.1. Chemical Composition and Nutritional Value of Faba Bean

Faba bean is a nutritionally valuable legume containing proteins, carbohydrates, dietary fibre, vitamins, minerals and various bioactive compounds. Carbohydrates make up the largest proportion of the seed dry matter, accounting for approximately 55–65%, with starch as the main component. The protein content of dry seeds ranges from 20 to 35%, while the fat content is generally low, at approximately 1–2% [6,7]. Seed composition may vary depending on genotype, growing conditions, stage of maturity and sample origin. Differences may also result from the analytical method used and the way the results are expressed [8,13,14].

Starch and proteins are mainly located in the cotyledons, the inner part of the seed, while the seed coat is richer in dietary fibre. Total dietary fibre in the whole seed may account for approximately 15–27% of the dry matter, with the insoluble fraction being predominant [8]. This distribution of components shows that dehulling is not only a preparation step, but also an important process that changes the composition of the raw material used for protein isolation. Removal of the seed coat reduces the fibre content, while the relative proportions of protein and starch increase. On the other hand, incomplete dehulling may result in higher levels of fibre and phenolic compounds in the protein preparation, which may affect the colour, taste and digestibility of faba bean products [6,15].

Faba bean also contains minerals such as potassium, phosphorus, magnesium, iron and zinc, as well as vitamins, particularly B vitamins and folates [1,13]. However, the total mineral content does not necessarily reflect the amount that can be used by the body. Phytic acid can bind certain minerals and thereby reduce their bioavailability. Therefore, the nutritional value of faba bean cannot be assessed solely on the basis of its chemical composition, but should also take into account interactions between individual components and changes that occur during processing [8].

In addition to its main nutrients, faba bean contains various phenolic compounds, saponins, phytosterols and other bioactive components. These compounds may contribute to antioxidant and other potential biological effects of faba bean products, but may also affect bitterness, astringency, colour and sensory acceptability [1,7]. The effects of these compounds depend on their concentration, chemical form and the processing methods used, and therefore they cannot be considered exclusively as undesirable components. Studies of total protein isolates and processing streams show that non-protein components are distributed differently among the fractions formed during processing. Consequently, the composition of an albumin isolate cannot be reliably predicted solely from the composition of the original seed [11].

### 3.2. Protein Content and Nutritional Quality of Faba Bean

According to the available literature, faba bean has a relatively high protein content, although reported values vary depending on genotype, growing conditions and the analytical method used. As shown in Table 3, the reported values indicate that faba bean contains more protein than chickpea, lentil, pea and common bean, but less than soybean and lupin [1].

Faba bean proteins contain all essential amino acids, but their proportions do not fully meet human nutritional requirements. They are particularly rich in lysine, an amino acid that is often limiting in cereal proteins. Therefore, combining faba bean with cereals can provide a more favourable overall amino acid composition. In contrast, methionine and cysteine are present in lower amounts and are generally the limiting amino acids in faba bean proteins [1,9]. The amino acid composition also differs between protein fractions, which will be discussed in more detail in the section on the albumin fraction.

The nutritional quality of proteins depends not only on their amino acid composition, but also on their digestibility. Protein structure, the presence of antinutritional compounds and processing methods can all affect digestibility. Appropriate processing can reduce the levels of protease inhibitors and other undesirable compounds and improve protein utilisation. However, intensive processing may cause changes in protein structure and reduce the availability of certain amino acids. Its effects therefore depend on the type and conditions of processing [6,8,9].

During digestion, faba bean proteins are broken down into peptides and free amino acids. Some studies have associated the resulting peptides with antioxidant, antihypertensive, anti-inflammatory and other potential biological activities. However, most available data come from in vitro studies and computational predictions, and it is still unclear to what extent these peptides remain stable, are absorbed and exert biological effects in the human body [1].

### 3.3. Protein Preparations Obtained from Faba Bean

Protein-enriched flours, concentrates and isolates can be obtained from faba bean. These preparations differ in their production methods, protein content and the amount of remaining non-protein components [12]. Protein-enriched flours are most commonly produced by dry fractionation, which involves milling and air classification of particles. During milling, smaller protein-rich particles and larger starch-rich particles are formed and their separation is based on differences in particle size and density [12,16]. The main advantages of this process are lower water and energy consumption and better preservation of the natural protein structure. However, the resulting protein-enriched fraction contains more starch, fibre and other seed components than concentrates and isolates [16].

Protein concentrates are obtained by partially removing non-protein components, while isolates are more purified preparations with a higher protein content. The limits used to classify these preparations are not fully standardised and different ranges can therefore be found in the literature.

Protein-enriched fractions obtained by dry processing generally contain approximately 40–65% protein, while values of approximately 53–71% have been reported for faba bean protein concentrates and 80–95% for protein isolates [12,17]. These ranges are based on the results of different studies and do not represent generally accepted regulatory limits. As the ranges partly overlap, preparations should be described by their actual protein content and production method rather than by product name alone.

Wet fractionation is based on transferring proteins into a liquid phase and then separating them from starch, fibre and other components. A common process involves extraction at alkaline pH followed by protein precipitation near the isoelectric point, which can produce concentrates and isolates with a high protein content [12,17]. However, the pH, temperature and drying method used can affect protein structure and properties, while some soluble components may be lost with the liquid phase [4,16]. Therefore, a higher degree of purification does not necessarily result in better nutritional or functional quality. The composition of the final product depends on the process used and the protein fractions retained during processing, which is particularly important when the aim is to obtain the faba bean albumin fraction [8].

## 4. Protein Fractions of Faba Bean

Plant seed proteins are traditionally classified according to their solubility in different solvents, following the Osborne classification. Albumins are soluble in water, globulins in dilute salt solutions, prolamins in aqueous alcohol solutions and glutelins in dilute acidic or alkaline solutions [12,18]. As this classification is based on the extraction method, the proportions of individual fractions may vary depending on genotype, sample preparation and the isolation conditions used [1,18].

Globulins are the main protein fraction in faba bean and consist mainly of storage proteins classified as 11S and 7S globulins. Legumin is the main representative of the 11S globulins, while vicilin and convicilin belong to the 7S globulins. Their proportions vary among genotypes and may affect the amino acid composition and functional properties of the proteins [8]. Warsame et al. [19] found that, across 35 genotypes, legumin accounted for approximately 50% on average, while vicilin and convicilin together accounted for about 27% of the quantified proteins. As these values were determined in extracted proteins, they cannot be directly considered as their proportions in the total seed protein. The remaining proteins include the less abundant albumin, glutelin and prolamin fractions.

## 5. Faba Bean Albumin Fraction

The faba bean albumin fraction comprises a heterogeneous group of proteins that share a common characteristic: water solubility. Unlike the predominant globulins, which mainly serve as storage proteins, water-soluble proteins include enzymes and proteins involved in metabolic and defence processes in the seed [8,19]. Therefore, the albumin fraction should not be considered a single protein, but rather a group of different proteins isolated based on their solubility. However, an aqueous extract does not necessarily contain only albumins, as other water-soluble proteins, including some globulins, may also be extracted [19]. To obtain a fraction of higher purity, proteins that do not belong to the target albumin fraction need to be removed after extraction. Isolation of the water-soluble fraction can produce an albumin-enriched protein preparation with a composition and properties that differ from conventional globulin-rich faba bean isolates [10,18]. Its characteristics depend on the composition of the isolated fraction and the extraction and purification conditions used [8].

### 5.1. Composition and Protein Profile of the Faba Bean Albumin Fraction

In some studies, albumins are reported as the second most abundant protein fraction in faba bean after globulins, accounting for approximately 12–23% of seed proteins [8]. This range describes the contribution of albumins to seed proteins and should not be interpreted as the protein content or albumin purity of an isolated preparation. However, the reported values depend on genotype, extraction conditions and the way the results are expressed. For example, albumin content may be expressed relative to total seed protein, extracted protein or the mass of the obtained fraction. Therefore, results from different studies can only be compared when the method used and the basis of calculation are taken into account [18,20].

Warsame et al. [19] analysed protein extracts from faba bean seeds using SDS-PAGE and mass spectrometry, rather than a separately isolated albumin fraction. They identified albumins, lipoxygenase, heat shock proteins, defensins and other less abundant proteins. Individual albumins were found in protein bands with molecular weights of approximately 10.2, 12.4 and 13.7 kDa. These results confirm the presence of low-molecular-weight albumins in faba bean seeds, but do not represent the complete protein profile of the albumin fraction isolated on the basis of water solubility.

A more direct insight into the composition of the isolated fraction was provided by Oluwajuyitan and Aluko [10]. They recovered the albumin fraction from a salt extract after dialysis and removal of precipitated globulins. SDS-PAGE was performed under reducing and non-reducing conditions, with the albumin preparation showing polypeptide bands of approximately 12–41 kDa under non-reducing conditions. Its profile was simpler than those of the globulin and glutelin fractions, but included several different polypeptides. The molecular weight ranges reported in these studies should be interpreted in relation to the different samples and analytical approaches used.

Luo et al. [21] isolated albumin fractions from ten legume species, including faba bean, by aqueous extraction followed by globulin precipitation and dialysis. The resulting fractions showed differences in their electrophoretic profiles among species. Low-molecular-weight proteins predominated in the albumin fractions, although protein bands with higher molecular weights were also present.

Taken together, the available studies describe the faba bean albumin fraction as a heterogeneous protein preparation containing several polypeptides, with low-molecular-weight components being prominent [10,19,21]. The exact profile varies with the sample and isolation procedure. Total protein content alone does not establish albumin-specific purity, because removing non-protein constituents can increase protein content without selectively enriching albumins. The recovered preparation should therefore be characterised by both its protein content and evidence of protein identity.

### 5.2. Isolation and Purification of the Faba Bean Albumin Fraction

**Figure 1:**
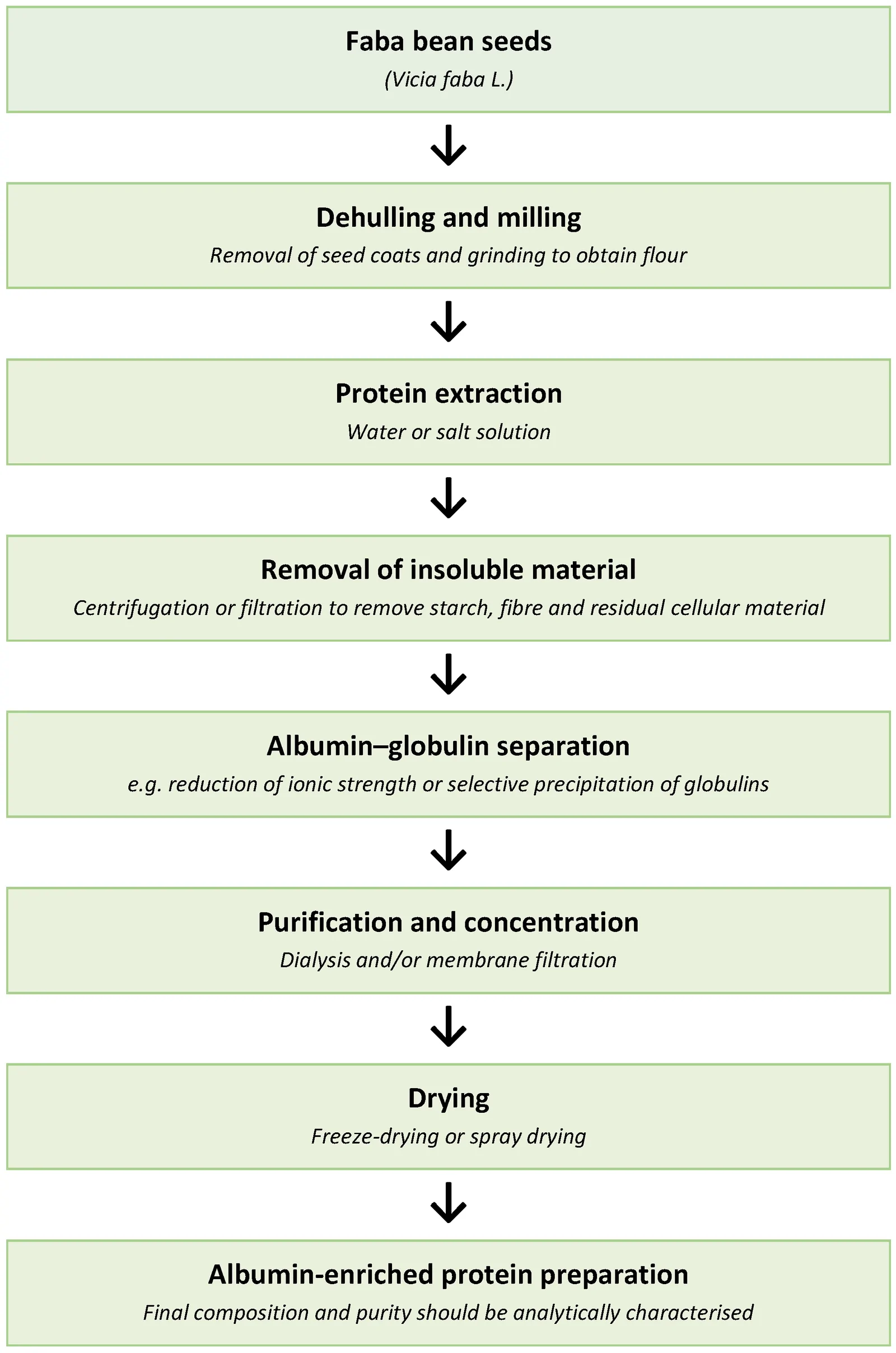
Schematic overview of the main steps in the production of a faba bean albumin isolate.

Available studies on the production of faba bean albumin isolates differ in the type of sample analysed. Some studies have isolated the albumin fraction separately, while others have examined albumin-enriched water-soluble fractions or total faba bean protein preparations. Results obtained from such preparations can help in selecting individual processing steps, but they do not directly confirm their effectiveness in producing an albumin isolate of known composition and purity. The main steps involved in the production of a faba bean albumin isolate are summarised in Figure 1.

The production of the albumin fraction begins with cleaning, dehulling and milling of the seeds. Dehulling removes the seed coat, which is rich in fibre and phenolic compounds, while milling increases the surface area. The preparation of the raw material can affect the yield and composition of the isolated protein fraction [6,12]. In a study of total faba bean protein isolates, Kamani et al. [22] found that dehulling contributed to higher isolate purity and solubility, while wet milling of whole seeds resulted in a higher yield. Although these results do not specifically refer to albumins, they show that raw material preparation should be selected according to whether protein recovery or the purity of the resulting preparation is prioritised.

The albumin fraction can be extracted using water or mild aqueous solutions because albumins are water-soluble. Extraction efficiency depends on the flour-to-solvent ratio, pH, temperature, extraction time and mixing intensity. A larger amount of solvent can improve protein transfer into the liquid phase, but it also increases the volume of extract that must later be concentrated. A longer extraction time does not necessarily lead to a proportional increase in protein yield [8,18]. Sengar et al. [20] used Osborne fractionation to separate protein fractions from the flour of eight faba bean varieties, with the albumin fraction isolated as the water-soluble protein fraction.

After extraction, insoluble starch, fibre and residual cellular material are usually removed by centrifugation or filtration, with centrifugation being used by Das et al. [18] in their laboratory-scale process. At a larger production scale, membrane processes can be used, although their efficiency may be limited by membrane fouling and the loss of some proteins in the permeate [11,23]. Centrifugation speed and duration can affect the yield and composition of the soluble fraction, so the separation conditions should be adjusted to the properties of the initial extract [18,24]. Bandyopadhyay et al. [24] showed that adjusting the separation conditions can partly change the albumin-to-globulin ratio in an aqueous extract. The resulting fractions were enriched in albumins but still contained globulin proteins, indicating that further purification is needed to achieve higher purity.

One method of separating albumins from globulins is based on changes in ionic strength. Oluwajuyitan and Aluko [10] dispersed dehulled faba bean flour at 10% (*w*/*v*) in a 2% (*w*/*v*) NaCl solution and stirred it for 240 min at room temperature. After centrifugation at 5600× *g* for 30 min at 4 °C, the supernatant was dialysed against distilled water for seven days at 4 °C using a 6–8 kDa molecular weight cut-off membrane, with three water changes daily. Subsequent centrifugation under the same conditions separated precipitated globulins from the albumin-containing supernatant. After freeze-drying, an albumin fraction containing 83.47% protein was obtained. This study provides direct evidence that this method can produce an albumin-enriched protein preparation. However, the reported 83.47% represents the total protein content of the preparation, rather than the proportion of albumins within those proteins, and therefore does not establish albumin-specific purity. The seven-day dialysis period also limits the use of this method at a larger production scale, while the relatively high ash content shows that inorganic constituents remained in the recovered preparation.

The albumin fraction can be further separated by removing proteins that precipitate at a selected pH. As part of Osborne fractionation, Sengar et al. [20] separated protein fractions based on differences in their solubility. Such separation is not necessarily completely selective, as the resulting fractions may contain proteins with different solubility properties. Exposure to strongly acidic or alkaline conditions may also cause changes in protein structure and promote aggregation, reducing subsequent protein solubility [8,20]. In a more recent study, Luo et al. [21] extracted faba bean flour with water at pH 8.0. After removal of the insoluble residue, the pH of the supernatant was lowered to 4.5 to precipitate globulins, while the albumin fraction remained in the supernatant. The supernatant was then dialysed for 48 h using a membrane with a molecular weight cut-off (MWCO) of 5 kDa, and the resulting fraction was freeze-dried. This method shows that isoelectric precipitation of globulins can be combined with dialysis to obtain an albumin fraction, although its suitability for larger-scale production is not yet known.

Membrane processes, particularly ultrafiltration and diafiltration, can be used for protein concentration and purification and may provide an alternative to lengthy dialysis. During ultrafiltration, protein retention depends on the membrane molecular weight cut-off and the properties of the proteins, while water and membrane-permeable solutes pass into the permeate. Subsequent diafiltration can further remove salts and other low-molecular-weight components [11,23]. Other protein fractions may also be retained, so additional separation may be needed to improve albumin-specific purity. Oluwajuyitan and Aluko [23] compared isoelectric precipitation and membrane ultrafiltration for the production of total faba bean protein concentrates. The membrane process resulted in a lower yield and protein content than isoelectric precipitation, but the protein concentrate had higher solubility. Although the study was not limited to albumins, the results suggest that membrane separation could provide a milder method for their concentration and purification.

The use of these membrane processes at an industrial scale requires careful consideration of protein recovery, product quality and operating costs. Pilot-scale membrane recovery of a soluble faba bean protein preparation has been demonstrated, although this does not establish an optimised process for a purified albumin isolate [11]. Membrane fouling and the associated decline in permeate flux can limit throughput and increase cleaning requirements. Studies of plant protein filtration have identified membrane blockage and cake formation as important fouling mechanisms [25]. The MWCO must also be experimentally optimised to retain the target albumins while allowing smaller unwanted constituents to pass, as membrane performance cannot be predicted from nominal MWCO alone. Research on soluble pea protein fractions demonstrates that MWCO influences the balance between protein recovery and removal of non-protein constituents, but the optimum cannot be transferred directly to faba bean albumins [26]. Economic assessment should therefore relate the costs of membrane investment, pumping, cleaning, replacement and diafiltration water to the mass of usable protein recovered. Mild operating conditions may favour native structure retention, but higher solubility alone does not demonstrate that proteins remain native [23]. Process selection should therefore balance protein recovery, structural integrity and operating costs rather than prioritising purity alone.

During aqueous extraction, non-protein components may enter the liquid phase together with soluble proteins. Their distribution during subsequent separation depends on their solubility and the processing conditions [11,27]. Of particular concern are vicine and convicine, which can remain in the same aqueous phase from which albumins are recovered. Collecting this phase without adequate purification may therefore leave these compounds in the final albumin-rich preparation. If these compounds are not adequately removed, subsequent concentration and drying may increase their concentrations in the final preparation compared with the initial aqueous extract. This is a safety concern because their aglycones, divicine and isouramil, can trigger favism in susceptible individuals with glucose-6-phosphate dehydrogenase deficiency, as discussed in Section 5.5 [11]. Lu et al. reported reductions of 98.5% in vicine and 99.7% in convicine during the production of a total faba bean protein isolate [27]. However, these reductions cannot be assumed for albumin isolation, which recovers proteins from the soluble phase. Krause et al. detected residual vicine and convicine in a protein preparation recovered by membrane filtration from the soluble fraction after isoelectric precipitation [11]. Thus, recovery of soluble proteins does not itself ensure removal of these compounds.

Ultrafiltration followed by diafiltration provides a possible route for separating these small compounds from soluble proteins without relying on thermal degradation. During diafiltration, fresh water is added to wash membrane-permeable compounds out of the protein retentate. Olsen and Andersen (1978) [28] found lower vicine and convicine contents in faba bean protein preparations obtained by diafiltration than in those produced by direct ultrafiltration. These findings support the inclusion of a washing step rather than reliance on protein concentration alone. However, the study examined faba bean protein preparations rather than a separately isolated albumin fraction, so the extent of glycoside removal and albumin recovery requires specific verification. Complete removal should therefore not be assumed from the use of a membrane process.

For an albumin-rich preparation, membrane selection and the extent of diafiltration should balance vicine and convicine removal against losses of smaller albumins. Operating at temperatures and pH values compatible with protein stability may help preserve albumin properties, but the absence of heat treatment does not itself prove retention of the native structure. Process evaluation should therefore include residual vicine and convicine concentrations, albumin recovery and protein structural or functional measurements. A higher protein content should not be interpreted as evidence of safety; residual glycoside concentrations in the final dried preparation must also be considered in relation to its intended use and the amount consumed.

The purified albumin fraction is finally dried to obtain a stable powdered preparation. Freeze-drying is often used in laboratory studies because it takes place at low temperatures and preserves protein structure relatively well, but it is time-consuming and expensive. Spray drying is more suitable for larger-scale production because it is faster and can be carried out continuously. However, the conditions used may cause protein denaturation and aggregation and alter protein solubility and functional properties [11,12,16]. Most data on the effects of spray drying refer to total faba bean protein preparations, and its effect on the properties of a separately isolated albumin fraction is therefore not yet known.

The possibility of scaling up faba bean protein extraction to pilot scale has been demonstrated using hydrodynamic cavitation [18], while membrane filtration and spray drying have been used in other processes for producing faba bean protein preparations [11]. However, these studies were mainly conducted on total proteins or albumin-enriched fractions rather than albumin isolates of confirmed purity. These processes can therefore be considered a starting point, but not a fully developed process for albumin isolate production. The main separation approaches, the preparations obtained and their principal limitations are summarised in Table 4.

Across these approaches, the common separation principle is to retain albumins in the soluble phase while removing insoluble material, globulins and unwanted membrane-permeable constituents. Salt extraction followed by dialysis and aqueous extraction followed by globulin precipitation have both yielded separately recovered albumin fractions [10,21]. Membrane processes offer a potential route to more continuous recovery and purification, although the available evidence also includes mixed protein preparations [11,23]. Process development should therefore focus on the balance between albumin recovery, selectivity, removal of undesirable constituents and preservation of protein properties. The available studies do not yet establish which approach achieves this balance most effectively at production scale.

### 5.3. Physicochemical and Functional Properties of the Faba Bean Albumin Fraction

The physicochemical properties of the albumin fraction determine its behaviour during processing and its potential use in food products. These properties depend on protein molecular weight, structure, surface charge and hydrophobicity, as well as on pH, temperature and the isolation method used. As the albumin fraction contains different water-soluble proteins, its properties may vary depending on the composition and purity of the resulting preparation [10,24].

The separately recovered faba bean albumin fraction examined by Oluwajuyitan and Aluko [10] showed polypeptide bands ranging from approximately 12 to 41 kDa. Far-UV circular dichroism measurements at pH 3, 5, 7 and 9 indicated that the secondary structure of the albumin fraction was dominated by unordered regions and β-sheet structures, with a relatively small proportion of α-helices. Beyond these secondary structural features, faba bean albumins may also have smaller particle sizes, a weaker negative surface charge and lower surface hydrophobicity [10,29]. Their smaller size may allow them to move more easily towards interfaces, while their weaker surface charge may reduce repulsion between protein particles and affect the stability of the resulting system [29].

Solubility is an important functional property because it is essential for the formation of stable dispersions, emulsions and foams. The faba bean albumin fraction showed higher solubility over a wider pH range than the globulin and glutelin fractions, with the highest solubility observed under alkaline conditions [10]. Shen et al. [29] also reported high solubility of faba bean albumins under the conditions studied. This may be an advantage in liquid and powdered preparations, although actual solubility will depend on the conditions of the final product, including pH, temperature, protein concentration and ionic strength. High solubility was also reported by Luo et al. [21], who compared the faba bean albumin fraction with albumins from nine other legumes. At a concentration of 3%, albumins from all legumes studied maintained solubility above 76% over a pH range of 3–9. However, solubility decreased as protein concentration increased, and at a concentration of 10%, the faba bean albumin fraction had a solubility of 68.86%. These results show that the high solubility observed in dilute systems cannot necessarily be expected in more concentrated products. In comparison, Barac et al. (2015) [30] reported minimum solubility near pH 5 for soy and pea protein isolates, with solubility increasing at more acidic and alkaline pH values. The broad solubility range reported for faba bean albumins therefore suggests a potential advantage in formulations where conventional soy and pea isolates show reduced solubility. However, this remains an indirect comparison because the preparations were examined at different concentrations and under different extraction and measurement conditions.

Results on thermal properties are not entirely consistent. Oluwajuyitan and Aluko [10] reported the onset of denaturation of the albumin fraction at approximately 77 °C, which was higher than that of globulins. Shen et al. [29] found similar denaturation onset temperatures for albumins and globulins, but a considerably lower denaturation enthalpy for albumins. These differences may result from the composition and purity of the fractions and the isolation and analytical methods used. Therefore, thermal stability cannot be assessed solely on the basis of the denaturation onset temperature.

The albumin fraction did not show the highest water- and oil-holding capacities, as glutelin had higher values in a comparative study [10]. This shows that good albumin solubility does not necessarily result in a greater ability to retain water or oil. These properties depend on the availability of hydrophilic and hydrophobic groups and on structural changes that occur during isolation and drying.

The emulsifying behaviour of the albumin fraction depends on its adsorption at the oil–water interface, the mechanical properties of the resulting protein film and the interactions between coated droplets. These factors can affect emulsion formation and storage stability differently. Adsorption at the interface should be distinguished from the formation of a mechanically resistant protein film, whose properties depend on interfacial packing and protein–protein interactions [29]. Shen et al. [29], who examined separately isolated albumin and globulin fractions from faba bean, lentil and chickpea, associated the small particle size and low denaturation enthalpy of albumins with dense interfacial packing and the formation of stiff protein networks that helped stabilise newly formed droplets during homogenisation. However, the albumin-stabilised emulsions remained susceptible to flocculation and coalescence during storage. The authors attributed this behaviour to insufficient electrostatic and steric repulsion associated with the low surface charge and small size of the albumin particles. Thus, forming a mechanically resistant interfacial film does not, by itself, ensure long-term emulsion stability. Surface hydrophobicity also contributes to interfacial behaviour. Bandyopadhyay et al. [24] linked higher surface hydrophobicity to faster adsorption and improved emulsion stabilisation in albumin-enriched water-soluble faba bean fractions. These preparations also contained globulins, so the observed effects cannot be attributed exclusively to albumins. For soy and pea protein isolates, Barac et al. (2015) [30] reported the lowest emulsifying activity near pH 5, where their solubility was also lowest. In contrast, the relatively high solubility reported for faba bean albumins across this pH region suggests that they may be useful for emulsion formation when soy and pea isolates are less soluble. This potential advantage still requires confirmation through direct comparisons, including measurements of emulsion stability during storage. Albumins can also contribute to foam formation through rapid adsorption at the air–water interface. Shen et al. [31] reported good foaming capacity and foam stability of legume albumin fractions, while Oluwajuyitan and Aluko [10] found higher foaming capacity for faba bean globulins at most of the pH values studied. These differences may be related to fraction purity, protein concentration, pH and the measurement method used. The results of Luo et al. [21] further show that the foaming and emulsifying properties of albumin fractions differ among legume species and depend on pH, which limits the extent to which these findings can be generalised to different formulations. Barac et al. (2015) [30] found that unheated soy protein isolates formed more stable foams than pea isolates across the pH values tested. Foam stability should therefore be considered when comparing faba bean albumins with soy and pea protein isolates, since rapid adsorption at the air–water interface alone does not ensure a stable foam.

The gelation ability of the albumin fraction has not been extensively studied. Oluwajuyitan and Aluko [10] found that a concentration of approximately 20% was required for albumin gel formation, while globulins and glutelins formed gels at 14%. Gelation was assessed in 0.1 M phosphate buffer at pH 7 after heating at 95 °C for 60 min, rapid cooling and refrigerated storage at 4 °C for 14 h. The least gelation concentration was determined by tube inversion, with gel formation recorded when the sample did not slip. This indicates a lower ability of albumins to form gels independently under the conditions studied. For comparison, Badjona et al. (2025) [32] reported a least gelation concentration of 10% (*w*/*v*) at pH 7 for commercial soy and pea protein isolates and an ultrasound-extracted total faba bean protein preparation. These samples were heated at 90 °C for 1 h and subsequently cooled at 4 °C for 12 h. Compared with the approximately 20% reported for the faba bean albumin fraction, these results suggest that albumins may require a higher concentration to form a self-supporting gel. However, differences in preparation composition and gelation protocols prevent a direct ranking of their gel-forming performance.

The smaller polypeptides and lower surface hydrophobicity of the albumin fraction may contribute to its higher gelation concentration by limiting the formation of sufficiently connected protein aggregates [10]. However, their contribution remains a possible explanation rather than an experimentally established mechanism. Disulfide cross-linking also depends on the accessibility and reactivity of sulfhydryl groups, rather than total sulfur-containing amino acid content alone. A shortage of available reactive groups should therefore not be assumed for faba bean albumins without direct measurements. Indeed, Shen et al. found that whole pulse protein extracts formed stronger gels than the corresponding globulin fractions and suggested that albumins contributed through disulfide bonds between aggregates, although starch and fibre also influenced the results [33]. Studies on total faba bean protein preparations further show that gelation depends on solubility, particle size, other constituents and the isolation method [33,34]. Their results therefore cannot be directly attributed to albumins.

For food texturisation, combining albumins with globulins is a promising approach to investigate, since limited independent gelation does not exclude a contribution to a mixed protein network [33]. However, the fraction ratio and processing conditions would need to be optimised in mixtures of defined composition. Enzymatic cross-linking with transglutaminase offers another prospective approach by forming covalent bonds between glutamine and lysine residues. Nivala et al. demonstrated increased gel stiffness following transglutaminase treatment of previously heated total faba bean protein isolate at neutral pH [35]. Its effectiveness for a separately isolated albumin fraction remains to be established. Both approaches should therefore be evaluated for gel strength, water retention and the protein concentration needed in the intended food formulation.

All of these properties are strongly influenced by the isolation method. Changes in pH and temperature may cause protein unfolding or aggregation, while drying may affect their redispersibility. Even mild aqueous fractionation can alter the albumin-to-globulin ratio in the soluble fraction and thereby affect its functional properties [24].

The clearest functional trend in the available studies is the relatively broad pH range over which faba bean albumin fractions remain soluble [10,21,29]. Their interfacial behaviour also supports investigation in emulsions and foams, although the ability to form these systems should be distinguished from their stability over time. Independent gel formation required a higher protein concentration than for globulins and glutelins in the direct fraction comparison [10]. These findings favour further evaluation in products requiring protein dispersion, while applications relying on stable interfacial films or gel networks may require formulation adjustments or combinations with other proteins. Direct comparisons under matched conditions are needed to establish any advantage over conventional plant protein isolates.

### 5.4. Amino Acid Composition, Nutritional Value and Digestibility of the Faba Bean Albumin Fraction

The nutritional value of a faba bean albumin isolate depends on its amino acid composition, protein digestibility and the availability of released amino acids for absorption. In a direct comparison of separately recovered faba bean protein fractions, albumins contained more sulphur-containing amino acids than globulins, suggesting a potential nutritional advantage. As globulins are the main protein fraction in conventional faba bean protein isolates, comparing these two fractions helps to assess the potential nutritional advantages and limitations of the albumin fraction and directly addresses the aim of this review. The amino acid compositions of the albumin and globulin fractions reported by Oluwajuyitan and Aluko [10] are presented in Table 5.

As shown in Table 5, lysine and leucine were the most abundant individually determined essential amino acids in the albumin fraction. The most pronounced difference between the two fractions was observed for sulphur-containing amino acids. Methionine and cysteine together accounted for 5.05% in the albumin fraction and 1.45% in the globulin fraction. The albumin fraction also contained higher proportions of lysine and tryptophan, but lower proportions of leucine and arginine. This composition suggests that the albumin fraction could nutritionally complement faba bean isolates in which globulins predominate. However, these results do not indicate that the albumin fraction has a generally higher nutritional value, as it did not contain higher proportions of all essential amino acids and protein digestibility must also be considered.

These values should be interpreted with caution because the protein composition of faba bean may depend on genotype and growing conditions, while the composition of the resulting fraction also depends on the selectivity of the extraction and purification methods used [1,19]. This is supported by differences between the available studies. As shown in Table 5, Oluwajuyitan and Aluko [10] reported a combined methionine and cysteine content of 5.05% in the faba bean albumin fraction. In contrast, Luo et al. [21] reported 2.74% cysteine and 0.65% methionine, corresponding to a total sulphur-containing amino acid content of 3.39%. These differences may result from the characteristics of the raw material, the composition of the resulting fraction and the isolation and analytical methods used. The observed differences therefore show that the amino acid composition of the albumin fraction cannot be considered a fixed characteristic, but should be interpreted in relation to the protein profile of the resulting preparation and the isolation method used.

A favourable amino acid composition alone does not ensure high nutritional value, as proteins must be digested and the released amino acids must be available for absorption. Oluwajuyitan and Aluko [10] estimated in vitro protein digestibility using a multienzyme pH-drop assay. Protein dispersions containing 6.25 mg protein/mL were adjusted to pH 8.0 at 37 °C and treated with trypsin, chymotrypsin and peptidase for 10 min. The estimated digestibility was 71.73% for albumins, compared with 75.34% for globulins and 77.33% for glutelins. These results provide a comparison of the fractions under the same assay conditions, rather than a direct measurement of amino acid absorption in humans. The authors associated the lower digestibility of the albumin fraction with its β-sheet-rich structure. In compact protein domains, close packing can restrict access to peptide bonds and hinder the positioning of susceptible sequences within the active site of digestive proteases. Proteolysis therefore depends not only on the presence of cleavage sites, but also on their exposure and the local flexibility of the protein chain. However, β-sheet content alone does not establish the accessibility of these sites, so this structural explanation should be regarded as a proposed mechanism rather than a demonstrated cause of the observed difference [10]. Protein aggregation and protease inhibitors or other constituents retained during isolation may also influence the measured digestibility [9]. Processing can either improve or reduce protein digestibility. Moderate heat treatment may partially unfold protein structures and inactivate heat-sensitive protease inhibitors, thereby improving the access of digestive enzymes. In contrast, excessive heating or exposure to extreme pH conditions may promote aggregation and reduce the availability of certain amino acids. Ayala-Rodríguez et al. [9] reported higher in vitro digestibility of heat-treated faba bean flour than of minimally processed flour. As these results do not refer to isolated albumins, it remains unclear to what extent the same effect of processing can be expected for an albumin isolate.

During digestion, albumin proteins are broken down into peptides and free amino acids. Studies of faba bean protein preparations have identified peptides with potential biological activities, but their origin specifically from the albumin fraction and their effects in humans remain unconfirmed [36,37]. Their potential relevance to food supplements is discussed in Section 6.2.

Future studies should complement static digestibility assays with dynamic or standardised semi-dynamic gastrointestinal models that account for changes in gastric pH, enzyme secretion and gastric emptying. Such approaches could clarify how processing and protein structure affect the rate and extent of albumin digestion [38]. Nutritional quality should also be evaluated using the digestible indispensable amino acid score (DIAAS), based on the ileal digestibility of individual indispensable amino acids and an appropriate reference amino acid pattern. DIAAS cannot be inferred from the overall in vitro protein digestibility percentage reported for the albumin fraction [39].

Taken together, the amino acid and digestibility findings identify a potential nutritional contribution of the albumin fraction, rather than an established overall advantage: its higher sulphur-containing amino acid content relative to globulins in the direct comparison was accompanied by lower estimated in vitro digestibility [10]. Further evaluation should establish whether this compositional difference translates into improved digestible amino acid provision in the final isolate.

### 5.5. Antinutritional Compounds, Allergenicity and Safety of the Faba Bean Albumin Fraction

When assessing the safety of a faba bean albumin isolate, it should be considered that some undesirable components may remain in the water-soluble fraction during protein isolation. These may include protease inhibitors and lectins, which are proteins and may therefore remain in the water-soluble protein fraction. Protease inhibitors reduce the activity of digestive enzymes, while lectins may interfere with nutrient absorption and adversely affect the intestinal epithelium [1,6,40]. These studies confirm their presence in faba bean and their potential adverse effects, but do not provide direct information on their levels or residual activity in a purified albumin isolate. Their presence and activity should therefore be determined separately in the final product. The activity of protease inhibitors and lectins can be reduced by appropriate heat treatment. Carbonaro et al. [40] found that only low levels of lectin and trypsin inhibitor activity remained after heating faba bean. However, intensive heating may cause protein aggregation and reduce protein solubility and digestibility. Processing conditions should therefore be adjusted to reduce the activity of antinutritional proteins while preserving the desirable properties of the albumin isolate as much as possible [12].

In addition to antinutritional proteins, the safety assessment of an albumin isolate should consider vicine and convicine, pyrimidine glycosides characteristic of faba bean [41]. As described in Section 5.2, because of the way the albumin fraction is isolated, the extent to which these compounds will remain in the fraction cannot be predicted in advance. Their levels should therefore be determined analytically in the final isolate. In individuals with an inherited deficiency of glucose-6-phosphate dehydrogenase, the breakdown products of vicine and convicine can cause oxidative damage to erythrocytes and an acute haemolytic reaction known as favism. Favism is not an allergic reaction because it does not result from an immune response to faba bean proteins, but from metabolic susceptibility associated with G6PD deficiency. Therefore, reducing the allergenicity of a protein preparation does not also eliminate the risk of favism [41,42].

Unlike favism, faba bean allergy is an immune-mediated reaction to its proteins. Clinical data remain limited, but cross-reactivity with proteins from other legumes is possible. Cutroneo et al. [43] found similarities between certain faba bean proteins and known legume allergens and detected IgE binding to components of the total extract, with the strongest reactivity associated with the legumin β-chain. These findings do not establish the allergenicity of a separately isolated albumin fraction. Although several major legume allergens belong to the 7S and 11S globulin families, low-molecular-weight proteins such as the peanut 2S albumins Ara h 2 and Ara h 6 are also important allergens [44]. Albumin enrichment should therefore not be assumed to produce a hypoallergenic preparation. Potential cross-reactivity with homologous legume proteins requires assessment, while distinguishing albumin allergens from globulins such as peanut Ara h 1 and pea Pis s 1 [43,44].

Future assessment should include IgE-binding assays performed specifically on the recovered faba bean albumin fraction using sera from clinically characterised patients with peanut or other legume allergies. Complementary immunoblotting and ELISA could identify IgE-reactive components and quantify binding, while inhibition assays would help distinguish cross-reactivity from independent co-sensitisation. The protein composition of the tested fraction should also be confirmed so that reactivity to residual globulins is not incorrectly attributed to albumins. Positive IgE binding alone would not establish clinical allergy; functional assays, such as basophil activation tests, would provide additional evidence of the biological relevance of the response.

Overall, the safety of a faba bean albumin isolate cannot be assessed solely on the basis of results obtained for whole seeds or total protein preparations. Its safety profile will depend on the composition of the actual isolate produced, including the retention of antinutritional compounds and potentially allergenic proteins.

## 6. Key Considerations for the Potential Applications of Faba Bean Albumin Isolate

The potential applications of faba bean albumin isolate depend on its nutritional value, functional properties and safety, as well as on the requirements of the intended product. The good solubility of the albumin fraction may be useful in liquid and powdered formulations, while its amino acid composition suggests potential use as an additional source of plant protein [10].

### 6.1. Food Applications of Faba Bean Protein Isolates and Implications for Albumin Isolate Use

Among the studies considered in this review, evidence on the use of separately isolated faba bean albumins in finished food products remains limited. Available research has examined the structural and functional properties of albumin fractions and their behaviour in laboratory systems, including model emulsions and foams [10,29,31]. Studies on total faba bean protein isolates therefore provide useful examples of food applications and highlight aspects that should be considered when evaluating an albumin isolate [45,46,47]. However, their findings cannot establish how the isolated albumin fraction would perform in the same formulations.

In bread, Maravić et al. (2024) [46] incorporated 7.2% faba bean protein isolate into a wheat–triticale flour formulation. Bread protein content increased from 9.63 to 13.1 g/100 g, while specific volume decreased from 3.38 to 2.83 mL/g. The bread also became harder, gummier and chewier. Sensory evaluation by ten expert panellists showed lower texture liking than for the control, although the bread remained acceptable and received favourable taste ratings. These results illustrate the need to balance protein enrichment with changes in bread volume and crumb texture.

A similar balance between nutritional composition and sensory quality was observed in pasta. Chan et al. (2019) [45] replaced 25% of durum wheat semolina with faba bean protein isolate, increasing protein content from 13.09 to 25.25 g per 85 g serving of dry pasta. Pasta formulations enriched with faba bean protein concentrate or protein isolate produced a modest reduction in postprandial blood glucose compared with semolina pasta. However, pasta containing the isolate received lower ratings for overall liking, taste and texture than the control. The authors associated the lower texture ratings with greater firmness under the fixed cooking conditions, suggesting that cooking time should also be considered when developing protein-enriched pasta.

Liquid formulations introduce additional considerations. Akintayo et al. (2026) [47] prepared vanilla-flavoured faba bean-based milk alternatives using an aqueous extract or 3 g of protein isolate per 100 mL of water. The isolate dispersion was hydrated at 50 °C for 50 min before further formulation and homogenisation. The isolate-based beverages also had a higher protein content than those prepared using the aqueous extract. To assess the effect of calcium fortification, 250 mg of calcium carbonate, equivalent to 100 mg of elemental calcium, was added per 100 mL of beverage. Isolate-based formulations had higher apparent viscosity than extract-based formulations, while calcium addition increased particle size and viscosity. Calcium-fortified beverages achieved moderate overall sensory acceptance, with mean liking scores of approximately 6 on a nine-point scale. However, the extract- and isolate-based recipes were independently optimised and differed in their oil, sucrose and flavouring contents, so differences between them cannot be attributed solely to the protein preparation. These findings illustrate the need to consider ingredient composition and mineral fortification together when developing protein-containing beverages.

Together, these studies show that the use of faba bean protein isolates requires attention to both nutritional composition and product quality. In bread and pasta, protein enrichment was accompanied by changes in texture and sensory acceptance, while the beverage study highlighted the influence of ingredient selection and calcium fortification on physical properties [45,46,47]. For faba bean albumin isolate, product trials are needed to determine incorporation levels that increase protein content while maintaining acceptable texture, flavour and processing stability. Comparing the albumin isolate with a total faba bean protein isolate in the same formulation would help establish whether fractionation offers an advantage in product quality or nutritional composition.

### 6.2. Applications in Functional Food Supplements

Food supplements are intended to supplement the normal diet with concentrated sources of nutrients or other substances with a nutritional or physiological effect [48]. In this review, functional food supplements refer to supplements in which the albumin isolate would provide protein and potentially additional nutritional or physiological value. Due to the solubility of the albumin fraction over a wide pH range, faba bean albumin isolate could be considered for use in powdered drink mixes, ready-to-drink protein beverages and other products dispersed in water [10]. When developing such a product, the recommended daily amount and the amount of protein provided by this intake should be defined.

The choice of product form and dose is also related to its acceptability. The characteristic taste and aroma of faba bean, colour, texture and sediment formation may limit regular product use, regardless of its protein composition [12]. The acceptability of an albumin isolate should therefore be evaluated in the actual formulation and at an amount corresponding to the intended daily dose. This should include ease of preparation, uniformity of dispersion, sensory acceptability and maintenance of quality throughout the intended shelf life.

The albumin isolate could also be investigated as a source of peptides with potential biological activity, released during digestion or controlled hydrolysis. Studies of faba bean proteins have reported antioxidant activity and inhibition of angiotensin-converting enzyme (ACE), an enzyme involved in blood pressure regulation [1,36]. However, activity measured in laboratory assays does not establish an antioxidant or blood-pressure-lowering effect in humans. Computational predictions likewise identify potentially active sequences without confirming their behaviour after consumption.

For a peptide to exert a systemic effect, it or an active digestion product must survive further breakdown, cross the intestinal barrier and reach its target at a sufficient concentration. Martineau-Côté et al. [37] examined some of these steps by combining simulated gastrointestinal digestion with a cell model of the intestinal barrier consisting of Caco-2 and mucus-producing HT29-MTX-E12 cells. Their findings demonstrate peptide transport under laboratory conditions, but do not establish absorption efficiency in humans or whether sufficient concentrations of active peptides would reach the circulation. Further breakdown in the body, including metabolism during the first passage through the liver, may change both the peptides and their activity. Moreover, these results were obtained from faba bean preparations rather than a separately isolated albumin fraction. Any proposed benefit of albumin-derived peptides therefore requires confirmation of their origin, stability during digestion and absorption, followed by evidence of efficacy in humans at the intended intake.

At present, faba bean albumin isolate can be considered a potential protein ingredient for food supplements, provided that the intended daily serving supplies a nutritionally meaningful amount of protein in an acceptable form.

### 6.3. Applications in Medical Nutrition

In this review, the term medical nutrition is used in a broader sense, while foods for special medical purposes denote products formulated for the dietary management of patients whose nutritional needs cannot be met by modification of the normal diet alone [49,50].

Faba bean albumin isolate could be considered as a potential plant protein source for such products, but its suitability cannot be assessed without a clearly defined target group. Protein requirements vary among patients and depend on the type and severity of the disease, nutritional status and ability to consume food. Nutritional support can increase energy and protein intake and improve certain nutritional and clinical outcomes in hospitalised patients who are malnourished or at risk of malnutrition [51]. However, these findings refer to complete nutritional support and do not demonstrate the effectiveness of albumin isolate as an individual ingredient.

For patients with reduced appetite or difficulties consuming food, it is important that the product provides a sufficient amount of protein in the smallest practical amount that can be consumed. Therefore, product development should consider how much protein is provided by the recommended serving and to what extent this intake meets the needs of the target group. Product volume, energy density and acceptability may affect regular consumption and consequently the achievement of the intended energy and nutrient intake [52].

Providing a high protein intake in a small product volume also introduces technological constraints. For oral nutritional supplements and enteral feeds containing albumin isolate, osmolality, viscosity and physical stability should therefore be assessed at the intended protein concentration. Residual minerals in the isolate should be characterised and accounted for when adding the mineral premix, as dissolved mineral ions can contribute to the osmolality of the final formula [53]. The ash content reported for the albumin preparation [10] indicates the presence of inorganic constituents, but does not establish their individual contribution to osmolality. The mineral composition and osmolality of the complete formula should therefore be measured. Retained salts may also modify the ionic environment and electrostatic interactions between proteins. Their effects should be evaluated together with pH and protein concentration when assessing aggregation and thermal stability [54].

This is particularly relevant for ready-to-use formulas subjected to ultra-high-temperature (UHT) processing. Nawaz et al. observed protein aggregation, droplet flocculation and coalescence in UHT-treated model beverages prepared with faba bean protein concentrate [54]. Although these findings do not establish the behaviour of isolated albumins, they highlight the need to assess viscosity changes, sedimentation and phase separation after the intended heat treatment and throughout storage. The final viscosity should be compatible with drinking or administration through the intended feeding tube; assessment of tube flowability in a plant-based enteral formulation provides an example of this practical evaluation [53]. For orally consumed products, bitterness, astringency and mouthfeel should also be assessed at the intended serving concentration. Consequently, the suitability of an albumin isolate for medical nutrition should be tested in the complete formulation, including its mineral composition, rather than inferred from solubility measurements in water alone.

Available data provide only an initial basis for assessing the faba bean albumin fraction. Oluwajuyitan and Aluko [10] determined its amino acid composition and in vitro digestibility, while Itkonen et al. [55] investigated the digestibility of cooked and dehulled faba bean in healthy individuals. The results of these studies cannot be directly applied to a purified albumin isolate or to patients for whom such a product might be intended. Its nutritional value and utilisation should therefore be confirmed using the actual isolate produced.

The tolerability of the final formulation should also be assessed when evaluating its suitability. This includes the ability to consume the recommended amount regularly without significant gastrointestinal discomfort or other adverse reactions. Such an assessment should be conducted in a clearly defined target group, as results obtained in healthy individuals cannot necessarily be applied to patients with specific nutritional needs. At present, faba bean albumin isolate can therefore be considered a potential protein ingredient for foods for special medical purposes, but its suitability for this application has not yet been confirmed. Before use, it should be established whether a product containing the isolate can provide the required amount of utilisable protein, whether it is acceptable and well tolerated and whether it achieves the expected nutritional effect in the target group.

### 6.4. Regulatory Requirements for the Use of Faba Bean Albumin Isolate

The regulatory requirements for faba bean albumin isolate depend on whether it is used in a food supplement or a medical nutrition product. Food supplements are intended to supplement the normal diet and are marketed in dose form. Foods for special medical purposes are intended for the dietary management of patients with specific nutritional needs and are used under medical supervision [48,49]. Therefore, the requirements for the use and labelling of the isolate must be determined according to the intended use of the final product.

Food supplements must state the recommended daily amount and the required warnings and the amounts of nutrients or other substances with a nutritional or physiological effect must be declared in relation to that daily amount [48]. Nutrition and health claims must comply with Regulation (EC) No 1924/2006. The claim ‘source of protein’ requires at least 12% of the product’s energy value to be provided by protein, while ‘high protein’ requires at least 20%. Health claims must be based on generally accepted scientific evidence; results from in vitro studies alone do not demonstrate a corresponding health effect in humans [56].

The formulation of a food for special medical purposes must meet the specific nutritional needs of a clearly defined group of patients, and its suitability for the intended use must be supported by generally accepted scientific data. The product must state that it should be used under medical supervision, while nutrition and health claims are not permitted [50].

The regulatory status of the albumin isolate should also be established before the product is placed on the market. The traditional use of faba bean does not necessarily mean that every selectively isolated protein preparation has the same regulatory status as the whole seed. However, the use of a new production process does not automatically mean that the isolate is a novel food. The assessment considers the history of use of the product in the European Union before 15 May 1997 and whether the production process may cause significant changes in composition or structure that affect nutritional value, metabolism or the level of undesirable substances. If the status of the product cannot be clearly determined after reviewing the available information, the food business operator may initiate a consultation procedure with the competent authority of the Member State in which the product is intended to be placed on the market for the first time [57,58].

Regardless of the regulatory status of the albumin isolate, its properties should be clearly defined and appropriate consistency between production batches should be ensured when assessing its quality and safety. The isolation method may affect the composition of the protein preparation and the levels of undesirable compounds, including phytic acid, lectins, vicine and convicine [11,27]. A product containing albumin isolate must comply with the general requirements for food safety and the relevant requirements for the provision of food information to consumers [59,60]. For its potential use in food supplements or foods for special medical purposes, specifications for the isolate should therefore be defined and its quality and safety assessed under the intended conditions of use.

## 7. Limitations of Current Research and Future Directions

Future research should distinguish properties of the albumin fraction from those arising from differences in preparation composition. Studies should report the isolation conditions, yield, total protein content and protein profile, and distinguish albumin-specific purity from overall protein enrichment. Consistent reporting would improve comparisons between studies and help identify processing conditions that preserve the desired nutritional and functional properties [1,8,10].

Another limitation is the small number of genotypes studied. The proportions of individual faba bean proteins are known to vary among genotypes, but such differences have not yet been sufficiently investigated for the albumin fraction [19]. Examining a larger number of varieties grown under different environmental conditions could help identify raw materials more suitable for albumin isolate production.

Most current knowledge of the digestibility and functionality of faba bean proteins is based on laboratory analyses and in vitro models. Such studies are useful for initial assessment, but cannot fully predict the behaviour of an albumin isolate during digestion or its effects in humans. Similarly, good solubility or other functional properties in model systems do not guarantee stability in a product containing other ingredients. The albumin isolate should therefore be tested in actual powdered and liquid formulations, with changes during processing and storage also being monitored.

Further research is needed to clarify the safety of the albumin isolate. As discussed in Section 5.2, wet fractionation can reduce the levels of certain undesirable components in total protein isolates because some of these compounds remain in the liquid phase after protein precipitation [27]. Their retention during albumin recovery from this phase therefore requires separate assessment. Likewise, allergenicity findings for total faba bean proteins do not establish the allergenic potential of the recovered albumin fraction [43]. Future studies should therefore include several independently produced batches, assess their consistency and relate the protein composition of the isolate to its nutritional, functional and safety properties.

## 8. Conclusions

Faba bean is a valuable but still underutilised raw material for the production of plant proteins. Available data show that albumins differ from globulins in their protein and amino acid composition and in certain functional properties. Their water solubility and the higher content of sulphur-containing amino acids compared with globulins, reported in a direct comparison of faba bean protein fractions, support further investigation of a separate albumin isolate. However, its suitability as an ingredient in food supplements and medical nutrition, and any advantage over existing protein ingredients, need to be demonstrated for the final isolate and its intended application.

Commercial development should proceed through three milestones. First, scalable purification processes should achieve adequate albumin recovery and removal of unwanted constituents while preserving native protein structure and functionality. Second, batch-to-batch consistency should be demonstrated against defined specifications for protein composition, residual antinutritional compounds and functional performance. Third, clinical studies in clearly defined target patient populations should establish tolerability and efficacy for the intended nutritional use. Successful commercialisation will depend on producing a consistent and safe albumin isolate that retains its intended functionality in the final product and delivers a demonstrated nutritional benefit to the target population.

## Figures and Tables

**Table 1:** Publication-year distribution of the scientific articles cited in the review.

Publication Year	Number of Articles
1978	1
2000	1
2015	1
2018	1
2019	3
2020	4
2021	6
2022	4
2023	5
2024	9
2025	11
2026	5
Total	51

European Union legislative documents and the FAO report were excluded.

**Table 2:** Main subjects and subsubjects of the scientific articles cited in the review.

Main Subject	Subsubjects	Number of Articles
Background and previous reviews	Faba bean composition and processing, phytochemicals, sustainability	8
Composition and nutritional value	Protein and amino acid composition, seed protein profiles, fraction composition	5
Extraction and fractionation	Dry and wet fractionation, Osborne Fractionation, extraction pretreatments, membrane purification	10
Physicochemical and functional properties	Protein structure, emulsification, foaming, gelation, thermal and enzymatic modification	8
Digestibility and bioactivity	In vitro and human digestibility, protein quality, peptide bioactivity, digestion methods	5
Antinutritional compounds and safety	Vicine and convicine, antinutrient reduction, favism, allergenicity	6
Food applications and clinical nutrition context	Pasta, bread, beverages, high-protein foods, oral supplements, enteral nutrition	9
Total		51

Each article was assigned to one principal category according to its main contribution to the review. Individual articles could address additional subjects. European Union legislative documents and the FAO report were excluded.

**Table 3:** Protein content of selected legumes, expressed on a dry matter basis. Source: Adapted from [1].

Legume	Protein Content (%) *
Chickpea	19.53
Lentil	22.15
Common bean	22.17
Faba bean	27.60
Pea	23.40
Soybean	40.00
Lupin	40.00

* The values presented are based on published data and may vary depending on genotype, growing conditions and the analytical method used.

**Table 4:** Comparison of selected extraction and purification approaches relevant to the recovery of faba bean albumins.

Approach	Preparation Obtained	Main Findings and Limitations	Reference
Salt extraction and dialysis	Separately recovered albumin fraction	Total protein content was 83.47%, but albumin purity was not established. Seven-day dialysis limits scale-up.	[10]
Aqueous extraction, Globulin precipitation and dialysis	Separately recovered albumin fraction	Albumins were recovered after extraction at pH 8.0 and globulin precipitation at pH 4.5. Scale-up suitability remains unconfirmed.	[21]
Mild aqueous fractionation	Albumin-enriched soluble fractions	Separation conditions affected the albumin-to-globulin ratio. Residual globulins require further purification.	[24]
Membrane separation	Mixed soluble proteins or total protein concentrates	Continuous processing may be feasible, but selectivity, protein losses, fouling and removal of undesirable constituents require evaluation.	[11,23]

The approaches were studied under different conditions and cannot be directly ranked. Total protein content does not establish albumin-specific purity.

**Table 5:** Comparison of the amino acid composition of the albumin and globulin fractions of faba bean. Source: Adapted from [10].

Amino Acid	Albumin Fraction (%) *	Globulin Fraction (%) *
Histidine	3.53 ± 0.02	2.69 ± 0.05
Serine	4.78 ± 0.02	5.22 ± 0.11
Arginine	5.45 ± 0.05	9.62 ± 0.02
Glycine	4.20 ± 0.01	3.41 ± 0.08
Asx ^1^	11.43 ± 0.01	12.10 ± 0.16
Glx ^2^	13.94 ± 0.08	19.53 ± 0.15
Threonine	4.23 ± 0.02	3.26 ± 0.03
Alanine	4.66 ± 0.05	3.67 ± 0.00
Proline	5.02 ± 0.05	4.43 ± 0.02
Cysteine	3.48 ± 0.12	0.78 ± 0.04
Lysine	8.75 ± 0.10	6.44 ± 0.04
Tyrosine	6.75 ± 0.09	4.00 ± 0.09
Methionine	1.57 ± 0.10	0.67 ± 0.01
Valine	4.90 ± 0.04	4.84 ± 0.03
Isoleucine	4.79 ± 0.05	4.75 ± 0.06
Leucine	6.48 ± 0.10	8.95 ± 0.03
Phenylalanine	4.24 ± 0.10	4.73 ± 0.04
Tryptophan	1.81 ± 0.04	0.91 ± 0.01
Sulphur-containing amino acids ^3^	5.05 ± 0.16	1.45 ± 0.03
Total essential amino acids	40.30 ± 2.21	37.24 ± 2.62

^1^ Asx represents the sum of asparagine and aspartic acid. ^2^ Glx represents the sum of glutamine and glutamic acid. ^3^ Sum of methionine and cysteine. * Values are expressed as mean ± standard deviation.

## Data Availability

No new data were created or analyzed in this study. Data sharing is not applicable to this article.

## References

[1] Martineau-Côté D., Achouri A., Karboune S., L’Hocine L. (2022). Faba bean: An untapped source of quality plant proteins and bioactives. Nutrients.

[2] Ortega R.M., Arribas-López N., Salas-González M.D., Aparicio A., González-Rodríguez L.G., Bermejo L.M., Lozano-Estevan M.D.C., Cuadrado-Soto E., López-Sobaler A.M., Loria-Kohen V. (2024). High-protein processed foods: Impact on diet, nutritional status, and possible effects on health. Nutrients.

[3] Madini N., Vincenti A., Beretta A., Santero S., Viroli G., Cena H. (2024). Addressing inflammaging and disease-related malnutrition: Adequacy of oral nutritional supplements in clinical care. Nutrients.

[4] Augustin M.A., Cole M.B. (2022). Towards a sustainable food system by design using faba bean protein as an example. Trends Food Sci. Technol..

[5] Duarte R.D.C., Iannetta P.P.M., Gomes A.M., Vasconcelos M.W. (2024). More than a meat- or synthetic nitrogen fertiliser-substitute: A review of legume phytochemicals as drivers of ‘One Health’ via their influence on the functional diversity of soil- and gut-microbes. Front. Plant Sci..

[6] Rahate K.A., Madhumita M., Prabhakar P.K. (2021). Nutritional composition, anti-nutritional factors, pretreatments-cum-processing impact and food formulation potential of faba bean (*Vicia faba* L.): A comprehensive review. LWT.

[7] Badjona A., Bradshaw R., Millman C., Howarth M., Dubey B. (2023). Faba bean processing: Thermal and non-thermal processing on chemical, antinutritional factors, and pharmacological properties. Molecules.

[8] Shi D., Stone A.K., Marinangeli C.P.F., Carlin J., Nickerson M.T. (2024). Faba bean nutrition: Macronutrients, antinutrients, and the effect of processing. Cereal Chem..

[9] Ayala-Rodríguez V.A., López-Hernández A.A., López-Cabanillas Lomelí M., González-Martínez B.E., Vázquez-Rodríguez J.A. (2022). Nutritional quality of protein flours of fava bean (*Vicia faba* L.) and in vitro digestibility and bioaccessibility. Food Chem. X.

[10] Oluwajuyitan T.D., Aluko R.E. (2025). Structural and functional properties of fava bean albumin, globulin and glutelin protein fractions. Food Chem. X.

[11] Krause M., Sørensen J.C., Petersen I.L., Duque-Estrada P., Cappello C., Tlais A.Z.A., Di Cagno R., Ispiryan L., Sahin A.W., Arendt E.K. (2023). Associating compositional, nutritional and techno-functional characteristics of faba bean (*Vicia faba* L.) protein isolates and their production side-streams with potential food applications. Foods.

[12] Sharan S., Zanghelini G., Zotzel J., Bonerz D., Aschoff J., Saint-Eve A., Maillard M.-N. (2021). Fava bean (*Vicia faba* L.) for food applications: From seed to ingredient processing and its effect on functional properties, antinutritional factors, flavor, and color. Compr. Rev. Food Sci. Food Saf..

[13] Dhull S.B., Kidwai M.K., Noor R., Chawla P., Rose P.K. (2022). A review of nutritional profile and processing of faba bean (*Vicia faba* L.). Legume Sci..

[14] Mayer Labba I.C., Frøkiær H., Sandberg A.S. (2021). Nutritional and antinutritional composition of fava bean (*Vicia faba* L., var. minor) cultivars. Food Res. Int..

[15] Khan F., Wilson R., Eyles A., Tivendale N.D. (2025). Enhancing the nutritional value of fava beans: Challenges and emerging strategies for minimization of antinutrients. Food Chem..

[16] Han R., Wang Y., Yang Z., Micklethwaite S., Mondor M., Paximada E., Hernández-Álvarez A.J. (2025). Industrial-scale fractionation of fava bean, chickpea, and red lentil: A comparative analysis of composition, antinutrients, nutrition, structure, and functionality. Curr. Res. Food Sci..

[17] Vogelsang-O’Dwyer M., Petersen I.L., Joehnke M.S., Sørensen J.C., Bez J., Detzel A., Busch M., Krueger M., O’Mahony J.A., Arendt E.K. (2020). Comparison of faba bean protein ingredients produced using dry fractionation and isoelectric precipitation: Techno-functional, nutritional and environmental performance. Foods.

[18] Das R.S., Zhu X., Hannon S., Mullins E., Alves S., Garcia-Vaquero M., Tiwari B.K. (2023). Exploring Osborne fractionation and laboratory/pilot scale technologies (conventional extraction, ultrasound-assisted extraction, high-pressure processing and hydrodynamic cavitation) for protein extraction from faba bean (*Vicia faba* L.). Innov. Food Sci. Emerg. Technol..

[19] Warsame A.O., Michael N., O’Sullivan D.M., Tosi P. (2020). Identification and quantification of major faba bean seed proteins. J. Agric. Food Chem..

[20] Sengar A.S., Alves S., Pojić M., Tiwari B.K., Tomičić Z., Banjac V., Tiwari U., Pathania S. (2025). Osborne fractionation and techno-functional properties of faba bean flours from eight varieties grown under a temperate maritime climate. Food Chem..

[21] Luo Y., Zhang Y., Zhang C., Li W., Simpson B.K., Rui X. (2026). Comparative assessment of albumins from ten pulses: Techno-functional properties and amino acid profiles for sustainable food applications. Sustain. Food Technol..

[22] Kamani M.H., Liu J., Fitzsimons S.M., Fenelon M.A., Murphy E.G. (2024). Determining the influence of fava bean pre-processing on extractability and functional quality of protein isolates. Food Chem. X.

[23] Oluwajuyitan T.D., Aluko R.E. (2026). Functional and physicochemical properties of fava bean protein concentrates obtained from isoelectric pH precipitation and membrane ultrafiltration. Appl. Food Res..

[24] Bandyopadhyay M., Nickerson M.T., Ghosh S. (2025). Effect of mild fractionation conditions on the composition, interfacial and emulsifying properties of water-soluble fractions from faba bean flour. J. Agric. Food Res..

[25] Arazo M., Jaque N., Caro N., Abugoch L., Tapia C. (2020). Development of a scalable procedure by a discontinuous crossflow DF/UF to obtain a concentrate of chenopodin from a dead-end centrifugal UF at bench scale. Food Chem..

[26] Chuchuca Moran G., Delgado Valverde C., MacRae S., Grossmann L. (2026). Ultrafiltration-driven refinement of soluble pea protein fractions reduces antinutritional load and increases protein content. Food Hydrocoll..

[27] Lu J., Zamaratskaia G., Langton M., Eriksson Röhnisch H., Karkehabadi S. (2024). Minimizing anti-nutritional factors in wet protein extraction from Swedish faba beans through the application of response surface methodology. Food Chem..

[28] Olsen H.S., Andersen J.H. (1978). The estimation of vicine and convicine in fababeans (*Vicia faba* L.) and isolated fababean proteins. J. Sci. Food Agric..

[29] Shen P., Twilt F., Nazir R., Peng J., Landman J., Sagis L.M.C. (2025). Role of globulins and albumins in oil–water interface and emulsion stabilization properties of pulse proteins. Food Hydrocoll..

[30] Barac M.B., Pesic M.B., Stanojevic S.P., Kostic A.Z., Bivolarevic V. (2015). Comparative study of the functional properties of three legume seed isolates: Adzuki, pea and soy bean. J. Food Sci. Technol..

[31] Shen P., Ha S.M.L., Peng J., Landman J., Sagis L.M.C. (2025). Role of pulse globulins and albumins in air–water interface and foam stabilization. Food Hydrocoll..

[32] Badjona A., Cherono B., Bradshaw R., Dubey B. (2025). Gelation and rheological properties of ultrasound-extracted faba bean protein: A comparative study with commercial plant proteins. Food Hydrocoll..

[33] Shen P., Ma X., Gouzy R., Landman J., Sagis L.M.C. (2025). Gelation properties of three common pulse proteins: Lentil, faba bean and chickpea. Food Hydrocoll..

[34] Tiong A.Y.J., Crawford S., de Campo L., Ryukhtin V., Garvey C.J., Batchelor W., van ’t Hag L. (2025). Legume protein gelation: The mechanism behind the formation of homogeneous and fractal gels. Food Hydrocoll..

[35] Nivala O., Nordlund E., Kruus K., Ercili-Cura D. (2021). The effect of heat and transglutaminase treatment on emulsifying and gelling properties of faba bean protein isolate. LWT.

[36] Asledottir T., Vegarud G.E., Picariello G., Mamone G., Lea T.E., Røseth A., Ferranti P., Devold T.G. (2023). Bioactive peptides identified in pea and faba bean after in vitro digestion with human gastrointestinal enzymes. J. Funct. Foods.

[37] Martineau-Côté D., Achouri A., Pitre M., Karboune S., L’Hocine L. (2024). Bioaccessibility and antioxidant activity of faba bean peptides in comparison to those of pea and soy after in vitro gastrointestinal digestion and transepithelial transport across Caco-2 and HT29-MTX-E12 cells. J. Agric. Food Chem..

[38] Mulet-Cabero A.-I., Egger L., Portmann R., Ménard O., Marze S., Minekus M., Le Feunteun S., Sarkar A., Grundy M.M.-L., Carrière F. (2020). A standardised semi-dynamic in vitro digestion method suitable for food—An international consensus. Food Funct..

[39] FAO (2013). Dietary Protein Quality Evaluation in Human Nutrition: Report of an FAO Expert Consultation.

[40] Carbonaro M., Grant G., Cappelloni M., Pusztai A. (2000). Perspectives into factors limiting in vivo digestion of legume proteins: Antinutritional compounds or storage proteins?. J. Agric. Food Chem..

[41] Khazaei H., Purves R.W., Hughes J., Link W., O’Sullivan D.M., Schulman A.H., Björnsdotter E., Geu-Flores F., Nadzieja M., Andersen S.U. (2019). Eliminating vicine and convicine, the main anti-nutritional factors restricting faba bean usage. Trends Food Sci. Technol..

[42] Luzzatto L., Arese P. (2018). Favism and glucose-6-phosphate dehydrogenase deficiency. N. Engl. J. Med..

[43] Cutroneo S., Calcinai L., Bertazzoli T., Ozcan D., Puxeddu I., Öztop H.M., Tedeschi T. (2025). Molecular characterization and allergenicity assessment of faba bean (*Vicia faba*) as protein ingredient. Appl. Food Res..

[44] Smits M., Verhoeckx K., Knulst A., Welsing P., de Jong A., Houben G., Le T.-M. (2021). Ranking of 10 legumes according to the prevalence of sensitization as a parameter to characterize allergenic proteins. Toxicol. Rep..

[45] Chan C.K.Y., Fabek H., Mollard R.C., Jones P.J.H., Tulbek M.C., Chibbar R.N., Gangola M.P., Ramadoss B.R., Sánchez-Hernández D., Anderson G.H. (2019). Faba bean protein flours added to pasta reduce post-ingestion glycaemia, and increase satiety, protein content and quality. Food Funct..

[46] Maravić N., Hadnađev M., Dapčević Hadnađev T., Pestorić M., Tomić J. (2024). Faba bean flour and protein isolate as partial substitutes in wheat-triticale bread: Technological and sensory properties. Food Feed Res..

[47] Akintayo O.A., Cormack O.G., Falconer R.J., Lauer J.C., Bozkurt H. (2026). Influence of faba bean extraction and calcium fortification on the physicochemical, structural, and sensory characteristics of faba bean-based milk. Food Chem..

[48] European Parliament, Council of the European Union (2002). Directive 2002/46/EC of the European Parliament and of the Council of 10 June 2002 on the approximation of the laws of the Member States relating to food supplements. Off. J. Eur. Communities.

[49] European Parliament, Council of the European Union (2013). Regulation (EU) No 609/2013 of the European Parliament and of the Council of 12 June 2013 on food intended for infants and young children, food for special medical purposes, and total diet replacement for weight control. Off. J. Eur. Union.

[50] European Commission (2016). Commission Delegated Regulation (EU) 2016/128 of 25 September 2015 supplementing Regulation (EU) No 609/2013 as regards the specific compositional and information requirements for food for special medical purposes. Off. J. Eur. Union.

[51] Gomes F., Baumgartner A., Bounoure L., Bally M., Deutz N.E., Greenwald J.L., Stanga Z., Mueller B., Schuetz P. (2019). Association of nutritional support with clinical outcomes among medical inpatients who are malnourished or at nutritional risk: An updated systematic review and meta-analysis. JAMA Netw. Open.

[52] Leon-Sanz M., Linares F., Gonzalo M., Tapia M.J., Maiz-Jimenez M., Ruiz Aguado M., Lizán L., Olveira G. (2023). Compliance with a high-protein and energy-dense oral nutritional supplement in patients with disease-related malnutrition: A randomized open-label crossover trial. Front. Nutr..

[53] Khamitova D., Saduakhasova S., Toimbayeva D., Kamirova Z., Taş N., Gökmen V., Kamanova S., Makangali K., Dautkanov N., Ospankulova G. (2026). Enzymatically Hydrolyzed Amaranth as a Base for Enteral Nutrition: Nutritional Characterization, Phenolic Bioaccessibility, and Physicochemical Properties. Foods.

[54] Nawaz M.A., Singh T.K., Stockmann R., Jegasothy H., Buckow R. (2021). Quality attributes of ultra-high temperature-treated model beverages prepared with faba bean protein concentrates. Foods.

[55] Itkonen S.T., Calvez J., Airinei G., Chapelais M., Khodorova N., Sahaka M., Benamouzig R., Stoddard F.L., Simojoki A., Pajari A.-M. (2024). True ileal amino acid digestibility and protein quality of 15N-labeled faba bean in healthy humans. J. Nutr..

[56] European Parliament, Council of the European Union (2006). Regulation (EC) No 1924/2006 of the European Parliament and of the Council of 20 December 2006 on nutrition and health claims made on foods. Off. J. Eur. Union.

[57] European Parliament, Council of the European Union (2015). Regulation (EU) 2015/2283 of the European Parliament and of the Council of 25 November 2015 on novel foods. Off. J. Eur. Union.

[58] European Commission (2018). Commission Implementing Regulation (EU) 2018/456 of 19 March 2018 on the procedural steps of the consultation process for determination of novel food status in accordance with Regulation (EU) 2015/2283. Off. J. Eur. Union.

[59] European Parliament, Council of the European Union (2002). Regulation (EC) No 178/2002 of the European Parliament and of the Council of 28 January 2002 laying down the general principles and requirements of food law, establishing the European Food Safety Authority and laying down procedures in matters of food safety. Off. J. Eur. Communities.

[60] European Parliament, Council of the European Union (2011). Regulation (EU) No 1169/2011 of the European Parliament and of the Council of 25 October 2011 on the provision of food information to consumers. Off. J. Eur. Union.

